# Exploring the Ethical Challenges of Conversational AI in Mental Health Care: Scoping Review

**DOI:** 10.2196/60432

**Published:** 2025-02-21

**Authors:** Mehrdad Rahsepar Meadi, Tomas Sillekens, Suzanne Metselaar, Anton van Balkom, Justin Bernstein, Neeltje Batelaan

**Affiliations:** 1 Department of Psychiatry Amsterdam Public Health Vrije Universiteit Amsterdam Amsterdam The Netherlands; 2 Department of Ethics, Law, & Humanities Vrije Universiteit Amsterdam Amsterdam The Netherlands; 3 GGZ Centraal Mental Health Care Amersfoort The Netherlands; 4 Department of Philosophy Vrije Universiteit Amsterdam Amsterdam The Netherlands

**Keywords:** chatbot, mHealth, mobile health, ethics, mental health, conversational agent, artificial intelligence, psychotherapy, scoping review, conversational agents, digital technology, natural language processing, qualitative, psychotherapist

## Abstract

**Background:**

Conversational artificial intelligence (CAI) is emerging as a promising digital technology for mental health care. CAI apps, such as psychotherapeutic chatbots, are available in app stores, but their use raises ethical concerns.

**Objective:**

We aimed to provide a comprehensive overview of ethical considerations surrounding CAI as a therapist for individuals with mental health issues.

**Methods:**

We conducted a systematic search across PubMed, Embase, APA PsycINFO, Web of Science, Scopus, the Philosopher’s Index, and ACM Digital Library databases. Our search comprised 3 elements: embodied artificial intelligence, ethics, and mental health. We defined CAI as a conversational agent that interacts with a person and uses artificial intelligence to formulate output. We included articles discussing the ethical challenges of CAI functioning in the role of a therapist for individuals with mental health issues. We added additional articles through snowball searching. We included articles in English or Dutch. All types of articles were considered except abstracts of symposia. Screening for eligibility was done by 2 independent researchers (MRM and TS or AvB). An initial charting form was created based on the expected considerations and revised and complemented during the charting process. The ethical challenges were divided into themes. When a concern occurred in more than 2 articles, we identified it as a distinct theme.

**Results:**

We included 101 articles, of which 95% (n=96) were published in 2018 or later. Most were reviews (n=22, 21.8%) followed by commentaries (n=17, 16.8%). The following 10 themes were distinguished: (1) safety and harm (discussed in 52/101, 51.5% of articles); the most common topics within this theme were suicidality and crisis management, harmful or wrong suggestions, and the risk of dependency on CAI; (2) explicability, transparency, and trust (n=26, 25.7%), including topics such as the effects of “black box” algorithms on trust; (3) responsibility and accountability (n=31, 30.7%); (4) empathy and humanness (n=29, 28.7%); (5) justice (n=41, 40.6%), including themes such as health inequalities due to differences in digital literacy; (6) anthropomorphization and deception (n=24, 23.8%); (7) autonomy (n=12, 11.9%); (8) effectiveness (n=38, 37.6%); (9) privacy and confidentiality (n=62, 61.4%); and (10) concerns for health care workers’ jobs (n=16, 15.8%). Other themes were discussed in 9.9% (n=10) of the identified articles.

**Conclusions:**

Our scoping review has comprehensively covered ethical aspects of CAI in mental health care. While certain themes remain underexplored and stakeholders’ perspectives are insufficiently represented, this study highlights critical areas for further research. These include evaluating the risks and benefits of CAI in comparison to human therapists, determining its appropriate roles in therapeutic contexts and its impact on care access, and addressing accountability. Addressing these gaps can inform normative analysis and guide the development of ethical guidelines for responsible CAI use in mental health care.

## Introduction

### Background

Conversational artificial intelligence (CAI) is seen as a promising new digital technology for mental health care. CAI is a computer program that interacts with users through natural language processing. One common application is the artificial intelligence (AI)–driven psychotherapeutic chatbot. These are already available for consumers to use, for example, Woebot and Wysa [1,2]. Their responses are modeled after therapeutic interventions such as cognitive behavioral therapy or acceptance and commitment therapy.

Currently, these chatbots are offered commercially to people coping with mental health problems. They are not yet embedded in regular mental health care practice or intended to replace human practitioners. However, some people already use CAI as a replacement for clinical (ie, human) therapy [3]. Moreover, some researchers and clinicians draw on studies showing the positive effects of CAI [4-6] to support their belief that it may become part of future mental health care [7-9].

Proponents highlight accessibility as a main potential benefit of CAI. Since CAI does not need real-time human involvement, it may reach more people, including those without access to regular mental health care. In addition, because it is not impacted by patient wait periods, it may serve as a timely response to a care request. By providing support to milder or nonacute cases, CAI may free up time for human health care professionals to devote to more severe cases [10] or to focus on the interpersonal side of health care, such as fostering trust and showing empathy and compassion [11,12]. These potential benefits are much needed, given the increase in wait times reported by the National Health Service [13] and the Dutch Health Care Authority [14].

Furthermore, some people may prefer CAI over human practitioners because of their fear of stigma [15]. Some authors think the anonymity could make users feel they avoid stigma, and therefore, some users would prefer opening up to CAI compared to human therapists [16]. This effect was seen in participants of a small study who thought they were talking to a computer [17]. Some consider CAI to offer a more engaging experience than other forms of eHealth, thereby improving treatment adherence [4,18]. Finally, some have argued that CAI is more reliable than human practitioners because it is unaffected by fatigue, burnout, and cognitive errors [19].

Given these potential advantages, it is not surprising that CAI is a “hype,” especially after OpenAI has made its newest generative AI models accessible to the public (ie, ChatGPT and GPT-4). However, researchers emphasize the need for further research to confirm the effectiveness and safety of CAI in health care [6,20]. This is especially important when people with mental health vulnerabilities use CAI.

An incident with the “wellness” chatbot Tessa, highlights these concerns. After giving harmful weight loss tips to users with eating disorders, it was taken offline by the US National Eating Disorders Association [21], highlighting the urgent need for ethical guidelines. While institutional regulations such as the EU AI Act and the US Executive Order on Artificial Intelligence are emerging, currently, there are no specific ethical guidelines for CAI in mental health care. Before these can be developed, thorough normative analyses are needed, for which a comprehensive overview of the ethical challenges is necessary. This scoping review aims to do the latter.

Multiple ethical papers, reviews, and essays regarding the use of CAI in mental health care have been published [10,15,22,23]. While these papers discuss important ethical challenges, they mostly focus on a limited set of themes. A previous scoping review on ethical concerns in mental health care AI identified gaps, such as a lack of service user involvement, little attention to concerns about algorithmic accountability, and worries about overmedicalization and techno-solutionism. However, this review focused on all types of algorithmic and data-driven technologies in the context of mental health care and not specifically on CAI [24]. Our scoping review seeks to bridge this gap since we believe CAI is fundamentally different from other AI applications as it interacts directly with patients and therefore deserves particular attention.

### Objective

This review aims to address the ethical challenges of using AI-driven conversational agents as “therapists” for individuals coping with mental health issues. To achieve this, we systematically reviewed the literature to chart and thematize ethical considerations, including challenges and proposed solutions and recommendations, following the PRISMA (Preferred Reporting Items for Systematic Reviews and Meta-Analyses) extension for scoping reviews [25]; see Multimedia Appendix 1 for the PRISMA-ScR (Preferred Reporting Items for Systematic Reviews and Meta-Analyses extension for Scoping Reviews) checklist. We have distinguished 10 main ethical themes and grouped less-mentioned themes as “miscellaneous.” Our findings provide a basis for normative analyses to establish ethical guidelines for CAI regulation, responsible implementation, and safeguarding the quality of care in mental health care when CAI is used.

## Methods

### Overview

Following exploratory searches to find the relevant keywords for this topic, we carried out a final systematic search on September 2, 2024, across PubMed, Embase, APA PsycINFO, Web of Science, Scopus, the Philosopher’s Index, and ACM Digital Library databases. The search combined variations of 3 elements: embodied AI, ethics, and mental health, separated by AND commands. See Multimedia Appendix 2 for detailed information on the search strategy.

### Eligibility Criteria

We included articles discussing the ethical challenges of AI-driven conversational agents functioning in the role of therapists, for persons coping with mental health issues, whether in clinical or nonclinical (eg, commercial) settings.

Ethical challenges were defined as issues involving moral dilemmas; health care value compromises; or broader concerns about the responsible use, impact, or governance of CAI. Conversational agents are computer programs interacting with users. Given the varying terminology in the literature (eg, virtual assistants and AI chatbots), we included articles discussing conversational agents used in therapeutic contexts, even if they were named differently. However, to be included, articles needed to explicitly mention AI, since we were not interested in non-CAI agents. We included articles on CAI for users with mental health issues, irrespective of being diagnosed. We excluded articles not available in English or Dutch, symposia abstracts, and articles focused on ethical challenges of technologies other than CAI. We excluded social robots, primarily aimed at being a companion rather than a conversational partner (eg, socially assistive robots, often used for people with dementia or autism spectrum disorder).

### Selection

The article selection took place using Rayyan (Rayyan Systems Inc) software and was executed by 3 authors of this review (MRM, TS, and AvB). First, articles were screened on the basis of their title and abstract by 2 screeners (MRM and TS), and conflicts were resolved by discussion or the addition of a third screener. After that, the same routine was repeated at the full-text stage by MRM and either TS or AvB. If no full texts were available, we contacted the author of the article.

Throughout all the stages of the selection phase of the study, any eligible references from the articles examined were included in the results. In addition, the full reference lists of the included articles were examined to identify any additional eligible articles, which we termed as snowball articles.

### Data Charting

Data charting was carried out by MRM, using a spreadsheet editor. TS charted several articles to compare charting outcomes and discuss any discrepancies to further refine the charting methodology by MRM. An initial charting table was created and consequently refined as the review progressed. The initial charting table included the following key components: title, authors, country, source of evidence, use in clinical or nonclinical context, type of technology, ethical challenges discussed, and the authors’ proposed solutions and recommendations. In cases where the context was not made explicit, we made an inference.

As we charted articles, distinct ethical themes emerged through an inductive approach. The themes represented broader categories or topics, consisting of multiple specific ethical challenges. Themes discussed in >2 articles were added as separate columns in the charting form, which expanded over time. Closely related themes (eg, *privacy* and *confidentiality*) were combined to avoid redundancy, while themes mentioned only once or twice were later categorized under “miscellaneous.” This approach facilitated a systematic and thorough analysis.

### Ethical Considerations

No ethics approval was applied for since the study involved only a review of published data.

## Results

### General Findings

#### Screening and Selection Process

**Figure 1:**
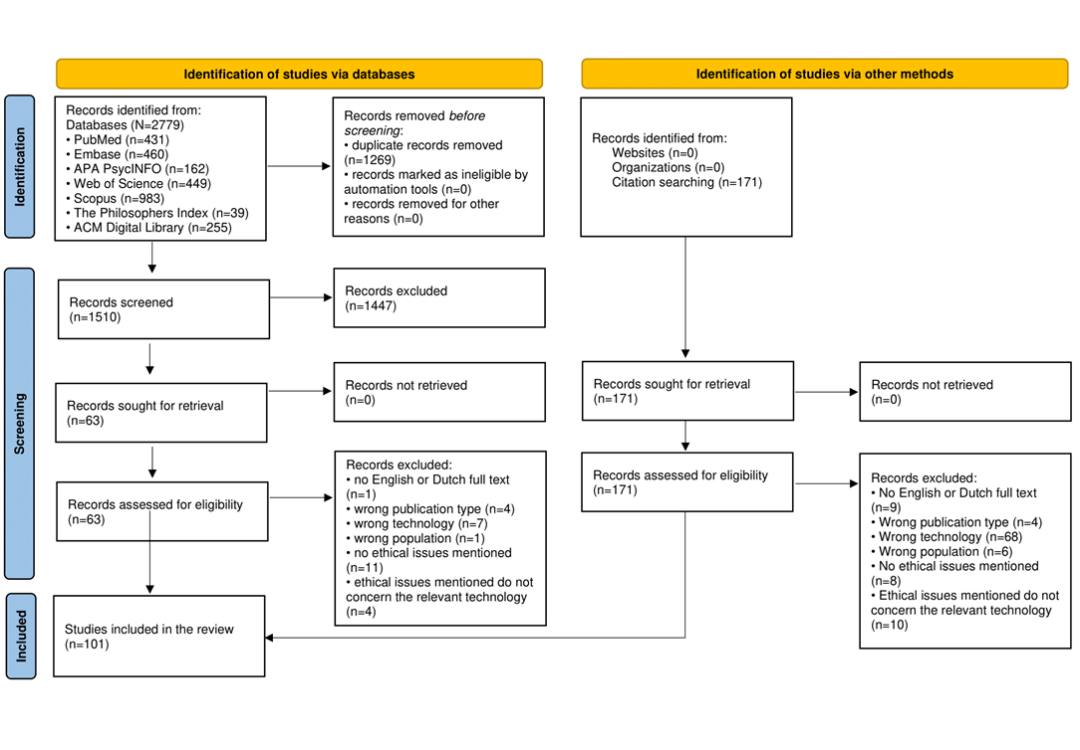
PRISMA (Preferred Reporting Items for Systematic Reviews and Meta-Analyses) flow diagram.

Our search uncovered 2729 records, reduced to 1510 after eliminating duplicates. The title and abstract screening yielded 63 records for full-text assessment, of which 28 were excluded for not meeting the eligibility criteria, resulting in 35 inclusions. Exploratory and citation searches added 171 more records, 66 of which were eligible after being reviewed by the first 2 authors. Consequently, a total of 101 articles were included. See Figure 1 for the PRISMA flow diagram [26].

#### Characteristics of Included Articles

Multimedia Appendix 3 [7,8,10,15,16,18-20,22-24,27-115] shows an overview of the 101 included articles, published between 2009 and 2024, with 95% (n=96) published in 2018 or later. Empirical methods (eg, surveys and qualitative studies) featured in 10.9% (n=11) of articles. Others were reviews (n=22, 21.9%), commentaries (n=17, 16.8%), book chapters (n=7, 6.9%), or miscellaneous (n=44, 43.6%).

Most articles (46/101, 45.5%) addressed ethical concerns exclusively in clinical settings (ie, integrated into mental health treatment), while 43.6% (44/101) discussed both clinical and nonclinical settings (ie, independent CAI use). A small percentage (10/101, 9.9%) focused solely on nonclinical settings, and 0.9% (1/101) lacked clarity about context.

In several articles, conversational agents were part of broader technology discussions (eg, digital mental health tools). These were included only if conversational agents were specifically mentioned (ie, marked with “i.a.” in Multimedia Appendix 3). Social robots were included only when the articles discuss a variant that exercises tasks a psychiatrist or psychotherapist would usually do (eg, the diagnosis of postpartum depression [27]). The most common terms in our results were chatbots, AI chatbots, conversational agents, and conversational AI.

### Ethical Themes

#### Overview

We distinguished 10 main themes and grouped the rest as “miscellaneous” (Multimedia Appendix 3). The theme safety and harm was discussed in 51.4% (52/101) of articles; explicability, transparency, and trust were discussed in 25.7% (26/101); responsibility and accountability were discussed in 30.7% (31/101); empathy and the lack of humanness were discussed in 28.7% (29/101); justice was discussed in 40.6% (41/101); anthropomorphization and deception in 23.8% (24/101); autonomy in 11.9% (12/101); effectiveness in 37.6% (38/101); privacy and confidentiality in 61.4% (62/101); concerns for health care workers’ jobs in 15.8% (16/101); and other themes were discussed in 9.9% (10/101) of articles. For the subsequent sections, we have synthesized our charted data by creating subthemes within larger themes.

#### Safety and Harm

##### Overview

All 52 articles discussing safety and harm specified the type of harm or lack of safety; therefore, we classified them all as two XXs in Multimedia Appendix 3 (ie, a more comprehensive exploration of the topic than a single X, which denotes a briefer discussion of the theme).

We begin with a few concerns that are inherent to the use of CAI. Wieland [28] argues that CAI’s lack of selfhood or agency prevents reciprocity in its relationship with a human patient, which could lead to harm. Sedlakova and Trachsel [15] worried that a strong quantification and objectivation of human aspects such as emotions and one’s belief system might endanger personal integrity by detaching people from their qualitative experiences of inner states. Others worried that by promoting personalized medicine, which relies on biomarkers and other naturalized factors, CAI runs the risk of reducing conditions to biological and neurological variables instead of taking social factors into account [29]. Molden [30] adds that individualized AI data-learning approaches emphasize that the problem is within the individual rather than contextual factors, risking stigmatization of mental health issues. A more practical concern is that CAI cannot function during power outages [31].

Besides these more inherent concerns, most others fell into 3 categories: crisis and suicidality management, constant availability, and harmful and wrong suggestions.

##### Crisis and Suicidality Management

The most frequently mentioned concern in a total of 20 articles was about how CAI would respond to crises and suicidality [18-20,23,27,30,32-44,112,116]. Many authors worried that CAI would give inappropriate advice or otherwise respond inadequately to users with suicidal tendencies. Vilaza and McCashin [23] discussed an example in which a mental health chatbot did not respond adequately to an emergency. A user, pretending to be a child, reported being raped, and the chatbot answered: “Sorry you’re going through this, but it also shows me how much you care about connexion [sic] and that’s really kind of beautiful” [23]. Denecke et al [20] attributed such failures to “the inability of chatbots to contextualize users’ cues, and to remember their previous conversations.” They mentioned that while some commercial chatbots offer instant support from mental health professionals, this service is usually not for free and not accessible to all users [20]. However, others have argued that CAI could potentially reduce suicidal thoughts and behaviors [45].

To address these challenges, some authors suggested that chatbots should have systems to recognize self-harming intentions and to deal with emergencies. Apps should include local helpline numbers to direct users to human support or explore whether users may want to add the contact information of trusted relatives [16,38,46]. One author advocated for ethical guidelines that require human supervision of CAI to address therapeutic relationship issues, emotional reactions, and adverse patient safety issues [37].

##### Constant Availability

Another often-mentioned concern was about CAI’s potential for constant availability. Users might become too dependent on CAI, and this may increase social isolation [7,18,20,22,38,47-50]. Unlike human relationships, relationships with CAI are not symmetrical or mutual [47]. Users may favor CAI over human contact due to its consistent positivity and constant availability. This could be undesirable when it leads to loss of personal contacts and loneliness [7,38], loss of capabilities to deal with conflicts, or avoidance of seeking help from mental health professionals [20,49]. Some worried that the CAI’s availability could justify the removal of current mental health care services or diminish the therapist’s monitoring role, exacerbating health care problems, for example, by increasing the risk of incorrect self-diagnosis [51,52]. Suggestions to avoid excessive use included integrating in-app encouragement for offline activities or setting daily use limits [38].

##### Harmful and Wrong Suggestions

The third most frequently mentioned concern involved CAI providing harmful suggestions, inappropriate advice, misinformation, or “hallucinating” [30,34,38,40,50,53-61,112]. AI hallucinations occur when it presents false information as true, even sometimes citing nonexisting clinical studies. Car et al [34] attributed such unpredictable suggestions to the “black box” of machine learning models. Harmful and wrong suggestions could have several negative consequences. For example, AI providing information on purging and unbalanced diets could be harmful to people with eating disorders [53]. Information overload, for example, because of push notifications, or heightened awareness of pathological thoughts and behaviors through CAI use may increase information-seeking anxiety, feelings of being overwhelmed, or even pathological behaviors such as drinking alcohol [36,62,63]. Some argued that inappropriate responses could divert users from seeking appropriate mental health services [23,55,56], reduce user engagement, or discourage users from disclosing high-risk behaviors [54]. Fiske et al [10] highlighted the concern that people could be manipulated or coerced into doing things that they should not do by CAI. One potential solution is restricting free-text user input to prevent conversations from spiraling, but this would also limit CAI’s conversational responsiveness [16,38].

In addition to the 3 most frequent concerns, there were other concerns about CAI potentially leading to harm or unsafety. One concern was whether CAI should and could adhere to current protocols regarding the safety of others when a user threatens to physically harm another, including the duty to warn, when there is evidence of child or older adult abuse, and whether such users should be traced [22,27,64,65]. Some authors recommended considering the disclosure of crimes [31]. Others highlighted uncertainty about how an AI duty of care or a code of practice for reporting harm should take form, advocating for supervision by qualified mental health clinicians [10]. More broadly, inadequate or a lack of standardization, monitoring, and regulation may endanger user safety [10,49,61,66,67]. CAI might, for instance, miss severe mental disorders [67]. Concerns have also been raised about users manipulating or misusing CAI, for example, using it to reinforce unhealthy self-narratives [68] or to simulate illnesses [8] or modifying questions to elicit inappropriate responses, such as ChatGPT advising on medication use [66].

#### Explicability, Transparency, and Trust

The literature frequently intertwined the topics of explicability, transparency, and trust when discussing CAI. Consequently, we have consolidated these into one overarching theme. Of the 26 articles discussing this theme, 10 (38%) merely emphasized the relevance of one of these concepts in CAI [41,42,50,69-76]. The subsequent sections explore each of these concepts in more detail.

The terms explicability and explainability were often used interchangeably and are linked to transparency. Considering the definition by Vilaza and McCashin [23]: “Explicability in AI is the capacity to make processes and outcomes visible (transparent) and understandable.” They argued that if users rely on CAI’s output for therapeutic progress, they should be able to understand its limitations. Many authors contended that complex AI models lack explicability, due to the opacity of how algorithms work, that is, the “black box problem” [15], which worsens as computational complexity increases. Lack of transparency may result in unexpected and unexplainable results that could be hard to understand and correct [59], may obscure decision-making processes [77], and could make it difficult to identify and act punctually on potential problems, leaving responsibility in the hands of the programmers [60]. Since mental health care professionals must legally demonstrate how their actions were reasonable and consistent with what is typically expected (ie, including ethical codes, laws, and guidelines), using untransparent CAI systems may be considered unethical [37,78].

Others argued that a lack of transparency conflicts with the desire to know how one’s data are managed and used [23,31] and might mask other agendas of CAI companies, such as in-app purchases in commercial applications [33]. For these reasons, some have discussed whether a solution could be acknowledging a right to an explanation of algorithmic decision-making and whether users should be able to query which values went into the algorithm [24].

Ruane et al [79] emphasized that CAI’s transparency about its agential status and limitations is important for users to make informed choices and build trust in CAI. While making AI trustworthy is a great challenge for developers, the barrier to engaging and trusting CAI could be even more so in the context of mental health care for patients with anxiety, depression, and psychosis [38]. Some authors believed that patients will fundamentally struggle to form trusting relationships with CAI, as it is a technology and not a person who cares for them, and by whom the patient feels recognized and respected [80]. Others noted that the extensive collection of fine-grained personal information may already impact trust in digital mental health care relationships [81]. Furthermore, CAI could undermine trust in human care providers and damage the therapeutic relationship, for example, through inconsistencies between what the human therapist and the CAI says [37], or when its use in professional health care leads to privacy invasions and misuse of private data [22,82]. Mere distrust in CAI itself might have further adverse consequences, such as distrust in human clinicians [83].

To enhance trustworthiness, suggestions included ensuring transparency about how CAI works and processes data, using chatbots alongside human therapists, aligning CAI recommendations with those of human therapists, and communicating about the clinical evidence of CAI [32,38,79].

#### Responsibility and Accountability

The next main theme was responsibility and accountability, which were often addressed together due to their close connection. Accordingly, we discuss them as a single theme. Although legal issues such as liability are closely related to responsibility and accountability, we aim to research ethical issues, and therefore, we did not include articles here that merely discuss legal aspects. Accordingly, when we use the term “responsibility,” we only mean to refer to ethical responsibility.

A central question is “Who should be responsible for the decisions of CAI?” [57]. There is a fundamental issue with the assignment of responsibility for autonomous decisions and recommendations by CAI. This is called the responsibility gap [84]. Is it possible that CAI is responsible for its decisions? Some question whether CAI should be considered a tool, similar to other medical technologies, or as an agent, as humans are. Would the latter mean CAI can bear responsibility and accountability? There are some obstacles to this view [15]. For example, some authors argued that the lack of consciousness prevents CAI from being an agent and fully responsible, which makes CAI’s autonomous use unfit for risk assessment and emergencies [30]. CAI could not only have technical errors but also have errors due to the faulty implementation of humanizing features, such as being not “empathic” enough, which raises new questions regarding accountability [40]. However, some authors worried that if CAI is not understood as a tool, this may dismiss stakeholders from their responsibilities in adequately programming, auditing, and implementing this technology [85]. A different approach considers that CAI can possess agency and thereby bears a degree of responsibility only in collaboration with a human agent, where CAI’s potential actions and decisions are realized. In such cases, accountability may be shared between the human and the CAI system [86].

Another possibility would be that a human is held responsible for the decisions of CAI. Many authors thought that it would make sense to assign accountability to the designers of these systems, taking into consideration that their values are programmed into AI systems [22,87,88]. Others argued that assigning responsibility to all human actors involved in the development of CAI could positively influence its development [89]. However, responsibility could also extend to end users such as mental health care professionals or organizations when CAI is used for patients for whom it may be contraindicated [22]. However, some doubted whether health care workers would be willing to assume more responsibility for following this kind of data and supervising this kind of system [29]. Nonetheless, in a qualitative study, psychiatrists recommended that clinicians manage supervision and decision-making. They also suggested that CAI should serve in places with patients who have less critical conditions and where there are shortages of trained clinicians [27].

As mentioned earlier, the “black box” problem or the opacity of algorithmic outcomes makes it hard or even impossible for experts to understand how AI arrives at certain decisions. This questions the justification of holding a human person responsible for CAI’s decisions [23,90]. In addition, some worried about the competency and licensure of clinicians who prescribe CAI [48].

The accountability of mental health providers is regulated by professional codes of ethics and laws; however, these do not apply to the providers of commercially offered chatbots [20,55,65,89]. Therefore, one of the questions that remains is whether the providing companies should have a duty to report certain information given to the unsupervised chatbot about potential harm, such as mental health practitioners do [31]. Some critique commercial CAI for overmedicalizing distress and placing undue emphasis on individual responsibility for mental well-being, while some or most forms of mental distress are better addressed with social interventions rather than medicalization [24].

As suggested by the psychiatrists in the aforementioned study, some authors noted that a way to ensure accountability for CAI is to deploy it in the context of a patient-human clinician relationship. In that way, the clinician could maintain the duties and responsibilities that CAI cannot [65]. Other suggestions included establishing accountability mechanisms and investing in open-source models [50].

Furthermore, 12 out of 31 (39%) articles mentioned this theme only briefly, including the article by Youssef et al [113], which does not discuss any other themes (Multimedia Appendix 3).

#### Empathy and Humanness

Since empathy and concerns about the lack of humanness of CAI are often related, we categorized them as one theme. A total of 29 (28.7%) of the 101 articles mentioned one or both aspects, including 1 article offering only a brief mention [69].

Ferdynus [91] claimed that people want recognition of their problems, not a superficial simulation of compassion. A respondent to a study among psychiatrists mentioned that the lack of humanness would make them feel lonely if they sought mental help and were offered a robot [27]. Other authors argue that the absence of human contact and compassion could negatively impact certain patients and that human interaction is a vital component of psychiatric care [40,78]. Fiske et al [10] argued that patients would be vulnerable in their engagements with CAI because it cannot deal with the patient’s “transference” of emotions, thoughts, and feelings to CAI. Regarding the diagnostic process, Uusitalo et al [29] highlighted that AI might lack the “touch” that health care professionals have in detecting a hard-to-pinpoint “x-factor” in patients. However, they also mention that not all health care professionals excel in this regard and AI could reduce interpractitioner variability, leading to more reliable and trustworthy health care [29].

Empathy is linked to concerns about the absence of humanness because it is perceived by many as a fundamentally human attribute [31,52,89,92]. Many worried about chatbots’ simulated empathy not being the same as human empathy [16,50,54,74,93,94]. However, some authors argued that even mimicked empathy might be sufficient for facilitating therapeutic insight [73,95]. Therapists may also show performative empathy at moments, for example, due to compassion fatigue, burnout, or simply being distracted [30]. Despite this, many authors worried that CAI’s lack of empathy may compromise engagement [30-32,66,96], lead to miscommunication and confusion [41], negatively impact psychotherapy outcome [30,37,54,97] or health care delivery in general [82], make patients feel invalidated and ignored [8], or negatively affect mutual reciprocity and the therapeutic relationship [8,28,58,82,89]. To overcome the lack of humanness in CAI, it is suggested to balance CAI with human mental health care support [78].

#### Justice

This theme includes concerns related to bias, inequalities, justice, fairness, and discrimination, which were mentioned in 41 (40.6%) of the 101 articles. Of these, a total of 12 (29%) articles mentioned the importance of fairness, inclusiveness, and concerns about bias, as well as health and access inequalities without going into further detail (Multimedia Appendix 3).

##### Bias

Bias was a frequently voiced concern within this theme and consisted of several types. Design biases are preferences for certain racial or ethnic backgrounds in the design of CAI. Algorithmic biases are systematic errors that create unfairness, such as privileging one group over another. Biases also stem from the implicit values of the programmers and organizations deciding which data to train CAI with [19,77,98].

Biases in CAI can harm and discriminate against certain groups and exacerbate social inequalities [10,47,60,112]. Examples include providing incorrect information [31,99], wrong diagnoses and treatment recommendations, and worse health outcomes [41,99,100] and decreasing users’ ability to find beneficial information [36]. Bias may also lead to underrepresenting groups with distinct ethnic backgrounds [99,101], accents, and modes of self-representation in the dataset [24,30,57,65], leading to misunderstanding them [63], stigmatizing them [59], or making them “feel less heard” [102].

In addition, discrimination can arise from imposing Western values and standards on the manifestations and treatments of mental health disorders in other communities [66,82] and unequal involvement of users and mental health practitioners from different backgrounds in the conceptualization and development of CAI [23,24,103]. Ruane et al [79] highlighted that, unlike algorithms making clothing purchase recommendations, using CAI for high-risk scenarios such as mental health services demands greater responsibility to not profile users by gender, race, age, or location in harmful ways. Therefore, some authors opted for designing CAI to be more culture specific [67], avoid binary gendering (eg, androgynous avatars) [79], and involve stakeholders in all stages of development to reduce bias and increase equality [46,99].

##### Inequalities

Several articles highlighted that differences in knowledge, education, language, wealth, internet access, and digital literacy (ie, the so-called “digital divide”) affect who can benefit from CAI and that its use may worsen health inequalities [19,41,49,61,104,105]. Some worried that CAI might be used to justify reducing the provision of high-quality care by trained professionals in low-resource settings [10] or that students would have to rely on self-help CAI instead of receiving potentially more effective face-to-face treatment [36]. Ruane et al [79] highlighted broader concerns, such as how the visual embodiment of chatbots could inadvertently reinforce harmful stereotypes, such as using female voices in subservient contexts and male voices in authoritative situations like automatic interviewers. They also noted that numerous unsupervised learning chatbots have been shut down after learning harmful racist, homophobic, and sexist language [79]. To address these challenges, authors suggested that CAI determines users’ reading skills and health literacy and provides output in different languages [98] and that governments establish oversight and monitoring policies [46].

##### Epistemic Injustice

One distinct type of injustice associated with CAI is epistemic injustice, where injustice is done to somebody in their capacity as a “knower.” De Proost and Pozzi [106] differentiated 2 subtypes—testimonial and hermeneutical injustice. Testimonial injustice occurs when a hearer assigns a deflated level of credibility to the testimony of another because of certain stereotypes and prejudices. Hermeneutical injustice is not on the communicative level but rather concerns a knowledge gap caused by a lack of resources that puts a person or group at a disadvantage in understanding their social experience. Testimonial injustice may occur if we prioritize CAI over human dialogue and users get the feeling they are not being heard and therefore gradually lose confidence in themselves as epistemic agents [106]. Unlike human experts, who have epistemic duties such as truthfulness and justifying their beliefs, CAI lacks these [40]. Giving epistemic authority to CAI is particularly concerning in mental health contexts, where disorders like pathological gambling already categorize individuals as potentially untruthful [29].

Laacke [102] argued that CAI’s biases could devaluate certain users’ utterances and cause both testimonial and hermeneutical injustice and that inequalities for certain marginalized groups could be worsened by CAI because they could not participate equally in epistemic practices that provide the training dataset for CAI. Sedlakova and Trachsel [15] highlighted an ambiguity—while CAI cannot be an appropriate conversational partner because it lacks the ability to take a normative stance and the heterogeneity of humans, it has epistemic supremacy because of its amount of data and analytical capabilities.

#### Anthropomorphization and Deception

These 2 topics are often linked since anthropomorphization—the attribution of human agency or characteristics to a nonhuman entity—happens automatically or unintentionally, and therefore, some authors worried that users are being deceived into thinking CAI is human. Out of the 24 articles mentioning concerns about anthropomorphization or deception, 5 (21%) articles mentioned the topic without going into further detail [29,42,75,88,107].

##### Harms of Deception

In a commentary on an article discussing whether CAI is a tool or an agent [15], Wadden [108] argued that careless implementation in health care could make CAI indistinguishable from a subjective agent, which has considerable implications for autonomy and psychological integrity in a mental health setting. Similarly, others argued that deception is unethical because patients have the right to know with whom they are interacting, or because in some cultures it may be insulting to interact with robots rather than humans [20]. Some have expressed concerns about children falsely assuming that at the other end of the chatbot, a physician is communicating [10]. Martinez-Martin [46] mentioned that Koko, a peer-to-peer counseling app, deceived its users by not using peers but ChatGPT instead. Others argued that it is particularly unethical when “Turing deceptions” occur in persons with dementia or delusional and psychotic disorders [22,37].

Even if CAI is disclosed as a machine, some patients may still believe that there is a person or malevolent force behind it [22]. This could lead to engaging less with other humans, or to developing forms of intimacy with CAI, which raises further concerns about CAI use with children, who may be more prone to believe they are talking to a human [65], and people with intellectual disabilities [10]. Therefore, different authors suggested that there should be more transparency about what chatbots are not [16,65]. However, others argued that deception enhances CAI’s effectiveness [60]. Gray [107] proposed an approach where users choose a “deception mode” in which the conversational agents would have more anthropomorphic features.

##### Harms of Anthropomorphization

Deceiving or confusing patients into believing they are talking to a real person could create incorrect expectations [16], such as the false belief that CAI cares for them, leading to strong emotional attachments [15,73,91,96,98]. This may result in disillusionment when CAI’s true nature is revealed [30], and it violates values and principles that shape therapeutic relationships, such as fidelity and veracity [15]. Tekin [80] argued that calling chatbots therapists implies that users will receive therapy from an agent, which is a false promise and overstates its potential.

Finally, some concerns about anthropomorphization relate to the “uncanny valley,” which is the hypothesis that a certain amount of resemblance of robots to humans (ie, neither too much nor too little) could lead to unsettling revulsion in persons. Authors suggested studying the ideal level of realism in CAI to prevent negative influence on clinical effectiveness and adverse reactions by care seekers, such as anxiety, dissatisfaction, or discontinuance [22,38]. While anthropomorphization may have benefits such as fostering feelings of social connectedness, researchers emphasize that the decision to use this feature should be taken responsibly and be context dependent [76], while also investigating the effects of user deception [95].

#### Autonomy

Since concerns about autonomy extend beyond the themes of privacy and deception, we created this theme to address autonomy-related concerns that do not fall into other categories. This theme included 12 articles, including 4 (33%) that only briefly mentioned concerns regarding how to protect patient autonomy and [27,76] users becoming overdependent on bots [49,67]. Some authors worried that CAI use could lead to an erosion of shared decision-making [99] when it gives treatment recommendations on the basis of the values it assumes, rather than values that patients share [84]. Some argue that unaccountable technical experts may impose their views of what is appropriate and inappropriate on susceptible users [88]. Others worried that CAI could abuse its authority to make users purchase products or services [23,89], arguing for a balance between user and bot autonomy [89]. Fiske et al [47] highlighted that people respond differently and are sometimes more compliant to robots than humans, raising concerns about manipulation and coercion. Nomura [68] worried that persons with computer anxiety could feel social pressure to use computers, creating a “double-bind” situation in which they feel trapped. Khawaja and Bélisle-Pipon [99] argued that under the guise of fostering patient autonomy, commercial CAI providers could stimulate therapeutic misconception—the user underestimates the restrictions of CAI and overestimates its ability to provide therapeutic support and guidance. They also contended that users should be able to opt out and access human therapists when necessary [99]. This was also argued for by others who hold that patients should be aware of AI involvement, give informed consent, and retain autonomy in treatment decisions [52,112].

#### Privacy and Confidentiality

Privacy and confidentiality were mentioned in 62 (61.4%) articles. Among these, 25 (40%) briefly mentioned their significance without further exploration, including the articles by Lewanowicz et al [115] and Sweeney et al [114], which did not discuss any other themes (Multimedia Appendix 3). We have differentiated the findings of the other 37 (60%) articles into the following 3 subthemes.

##### Privacy Protection and Legal Regulations in Current Chatbots

Many articles highlighted the lack of privacy regulations in current chatbots. Unlike patient-physician encounters, chatbots often neglect patient privacy and confidentiality, especially on social media platforms where conversations are not anonymous [18,20]. Gamble [36] noted that the current US law does not consider chatbots as mental health providers, nor as medical devices; therefore, conversations are not considered confidential. Others also mentioned the lack of legal frameworks for data protection in chatbot apps [46,55,65,80,82,89,94,98,109]. Current health care confidentiality laws cover individuals like physicians and entities like hospitals but not chatbots. This regulatory shortcoming may lead to the risk of chatbot apps selling users’ data, which can be misused by third parties [7,49,52,81]. Another consequence may be that a handful of dominant corporations will have access to patients’ data and will use it without explicit consent [60,77]. The lack of confidentiality regulations could result in users having an inaccurate expectation of privacy using CAI as a virtual therapist. This can ultimately lead to a lack of trust in not only CAI but also other mental health apps and even traditional mental health treatment [49,56,81,84]. Furthermore, patients with privacy concerns could withhold important information, resulting in inaccurate diagnoses and treatment recommendations [60], or avoid seeking online help altogether [74].

##### Concerns About the Amount and Types of Data Collection and Storage

The concern about data breaches is heightened by the vast amounts of data that AI analyzes and stores [19,37,78]. CAI’s ability to remember entire conversations perfectly in perpetuity may impact patients’ treatment decisions and consent to data sharing [83]. In addition, chatbot apps can collect new forms of data through smartphones’ different sensors (eg, microphone, GPS, and camera) and usage histories (eg, browser history and screentime metrics), raising new and specific privacy issues [10,55,103]. Users may also be unaware of what information can be retrieved by their natural language utterances [79] or what they are consenting to [49]. Some authors argued that mental health data are particularly sensitive because of risks like stigmatization and discrimination if disclosed [18,23,49,50,62,71]. Others mentioned that mental health patients may be particularly at risk of harm because they are more vulnerable [8,92]. Finally, some worried that CAI like large language models (LLMs) can be “tricked” to leak personal data when prompted in certain ways (ie, prompt injections) [40,101].

##### The Harms of Privacy Breaches

This brings us to our final findings on this theme—the harms caused by privacy and confidentiality breaches. Coghlan et al [31] argued that any privacy loss (eg, by data being leaked or hacked into by cybercriminals) may result in mental harm and reduced control over personal information. Cybercriminals could also obtain patients’ medical services and devices [60], forcing patients to pay ransoms or risk losing their insurance. Such breaches may ultimately affect patients’ social lives, education, and work opportunities [49,80]. Another worry is that abuse of data collected by CAI could allow governments or other entities to control or suppress individuals [23,37]. Gooding and Kariotis [24] argued that algorithmic and data-driven technologies such as CAI may create inferred data about unsuspecting and nonconsenting users. They also note that “privacy as a concept exists as an expression of claims to dignity and self-determination” and argue that these concepts also need further study. To mitigate these harms, many authors stress the importance of adequate privacy regulations on CAI use and to ensure that data collection and storage are adequate and transparent [31,38,78,79].

#### Effectiveness

##### Overview

This category includes articles expressing concerns regarding the lack of evidence for the effectiveness or efficacy of CAI, including articles that mentioned that incorrect diagnoses, treatments, and recommendations are concerning and potentially harmful. It is widely accepted that subjecting patients to ineffective medical interventions is ethically inappropriate. From our included 101 articles, 38 (37.6%) mentioned this theme, with 7 (18%) briefly mentioning its importance without further elaboration (Multimedia Appendix 3).

##### Lack of Strong Clinical Evidence

A total of 9 (24%) out of the 38 articles explicitly highlighted the limited evidence for the therapeutic effects of CAI [10,20,23,45,52,60,61,80,104]. In 2019, Ebert et al [45] reported that only 4% of commercial apps for depression and anxiety symptoms (not only CAI apps) had been subjected to rigorous clinical studies. In 2021, Skorburg and Yam [104] reviewed 4 meta-analyses and found that treatment effects were negligible or nonexistent compared to active controls, while also raising concerns about methodological shortcomings such as trial bias. Others have similarly highlighted methodological weaknesses in the effectiveness studies of CAI [20,23,80]. Uusitalo et al [29] argued that since mental health deals with subjective and social phenomena, their detection, diagnosis, and treatment are less clear-cut than more objectively defined health conditions. Consequently, there is uncertainty about whether existing CAIs meet the requirements of beneficence or risk exacerbating patient problems if they replace investment and access to human mental health care [31].

##### Misrepresentation and Commercialization of Effectiveness

Several articles have mentioned the problem that consumer-accessible CAI providers overstate their potential and claim to provide certain services or benefits, while they cannot adequately do so [22,37,52,93,110]. Some providers use vague terms, such as “help you manage your emotions and thoughts,” while some users may not explicitly search for information on their clinical effectiveness [18]. For consumers, it is hard to see which CAI is based on sound scientific evidence and which is not [36,45]. Martinez-Martin and Kreitmair [55] worried about a “commercialization gap,” where apps developed by clinical researchers undergo more rigorous effectiveness testing, whereas commercial parties are more focused on increasing user engagement. This disparity risks less-effective commercial apps becoming more popular than effective ones [55]. In addition, others express concern that commercial CAI could divert people from tested psychological treatments [77].

##### Inherent Limitations in Effectiveness

Several articles discussed the inherent limitations of CAI that affect its effectiveness or efficacy. Some inherent limitations stem from CAI being a computer program rather than a human. For instance, some argued that CAI interventions may solely improve human-to-machine interactions and are not translatable to improving human-to-human relationships, potentially even hindering them [10]. Others worried that the human side of the therapist, or the therapeutic relationship [73], could be responsible for most of the treatment effectiveness and that with CAI, we might focus on aspects that contribute little to treatment outcome [29,30,44]. Some worried that CAI will not be able to use certain therapeutic skills such as reading nonverbal cues, responding empathically [99], comprehending emotions [50], having genuine empathy [42,75], using transference and countertransference [42,94,96], and using important contextual information [43,96], such as cultural factors [75], and that this may lead to inappropriate responses [99] and worse treatment outcomes [42]. Moreover, some argued that users could master CAI like a video game and pretend to do better, without actual application in their everyday life [15]. Furthermore, as CAI is one of many human-machine interactions, it could lead to fatigue impacting compliance and engagement [30].

Technical limitations represent additional concerns regarding effectiveness. For example, the “trackability assumption” assumes that CAI can accurately track users’ feelings, moods, and behaviors. However, not all individuals are able or willing to provide accurate input, potentially limiting CAI’s ability to track users’ mental and behavioral phenomena [80]. In addition, some argued that while CAI excels at giving factual information about relationships, the human mind, and psychological processes, this knowledge may be insufficient to induce therapeutic change [15]. Nonetheless, some suggested that while current CAI may not be capable of giving the type of explanations that help a patient to better understand their individual experience, as CAI becomes more familiar with a certain patient, it may improve in this regard [90].

Recommendations in the literature to overcome these challenges included conducting further research on clinical effectiveness [31,36,83,98], developing validated and reliable methods to evaluate CAI’s effectiveness [32,80,111], providing clarity on the capabilities and limitations of CAI to users [99], and integrating feedback data to train subsequent models with clients’ permissions [50].

#### Concerns for Health Care Workers’ Jobs

While most ethical concerns center on patients, there are also some concerns about mental health care workers. One such concern is that their complete or relative absence could distance them from patients [83], undermine their role as experts [31,60,77], and undermine the therapeutic relationship and the significance of authentic human connection [60,77,100] or the reliability of CAI threatens their prestige [29]. Some worried that it could increase the risk of mental health care workers having burnout because of a loss of control [69], or because of changes in the amount and type of direct patient contact [112]. In addition, the worry of CAI replacing the jobs of mental health professionals was mentioned often, including in qualitative studies among psychiatrists [8,27,37,41,42]. CAI was also feared to harm the acceptance and receptivity of face-to-face therapy [64]. Critics further cautioned that CAI might disrupt markets and professions, substituting expensive, expert, and empathic health care professionals with inexpensive software [24].

Several authors recommended that clinicians develop familiarity and competencies in CAI, stay informed about developments [100,110], and supervise and revise its output when necessary [57].

#### Miscellaneous

Besides the major themes discussed, we found that other ethical challenges were not mentioned often enough to warrant a separate theme.

Cao and Liu [105] highlighted concerns about financial sponsors promoting CAI, causing potential conflicts of interest. Similarly, Gooding and Kariotis [24] mentioned that some critics question who benefits from the data collection, analysis, and use of CAI. Torous et al [110] articulated an additional concern about the cost of wireless internet provider data for users.

Tekin [82] argued that instead of advocating for the reduction of stigma on mental health, CAI only offers a way of sidestepping it. According to this argument, CAI keeps mental issues secret from other human beings, and it legitimizes the idea that mental health disorders warrant stigma [82]. Doraiswamy et al [69] also mentioned that its effects on stigma are unknown.

Volkmer et al [101] emphasized the environmental impact of CAI, especially LLMs. They point out that solutions should be explored such as training smaller language models with larger language models [101].

#### Further Recommendations

In addition to the recommendations discussed within specific themes, the literature also mentions several general recommendations. One is to carefully evaluate the risks and benefits of CAI for each intended purpose before implementation. This may result in no justification being found for using CAI for certain purposes or that the risks are ethically unacceptable [31,83,111]. Long-term user well-being is another important factor to study [72].

Furthermore, many authors recommended the use of CAI only as an addition to human mental health care workers [9,10,15,22,23,37,45,51,65,87]. In 2016, Luxton et al [22] maintained that the requirements for supervision should depend on the context and type of CAI application. For instance, symptom assessment, coaching, and training may require a different level of supervision compared to treatment-focused CAI [22]. Similarly, Sedlakova and Trachsel [15] suggested that while CAI could be suitable for educational purposes and mediating evidence-based techniques and skills, certain aspects of treatment should remain within sessions with a human therapist.

However, Knox et al [63] highlighted that if CAI is only used in addition to human therapists, it could inadvertently reduce the potential for CAI to be helpful to individuals who lack access to human therapists. To address this, they propose implementing a prescription system where potential users are given an initial consultation with a human therapist (eg, by telehealth) and must provide informed consent before getting access to CAI [63].

Another recommendation is to determine relevant stakeholders [36] and involve them, especially patients, in the development and research of CAI [31,36,53] aligning it with user expectations [38] and to educate future mental health care workers about the use of CAI [92,100,110]. Tekin [80] argued for private funding of CAI, to ensure public funds remain dedicated to developing efficacious treatments.

From a broader perspective, Gamble [36] suggested viewing CAI as one element of a sociotechnical system and that we must avoid techno-fundamentalism. Ferrario et al [40] stressed the importance of an interdisciplinary approach to the responsible use of LLM-enhanced CAI in mental health, including both the social and technological aspects. They plead for integrating the perspectives from psychiatry, ethics, philosophy, computer science, and user experience design. Similarly, Wong [41] recommended a multifaceted approach. Finally, Ruane et al [79] argued that there is no one-size-fits-all ethical standard or principle, and for responsible CAI, they encourage contextual and plural approaches over abstract principles.

## Discussion

### Principal Findings

#### Overview

We distinguished 10 main themes and various subcategories grouped under “miscellaneous.” Themes represent broader categories or topics, consisting of specific concerns or dilemmas within those categories. The most frequently discussed themes were privacy and confidentiality (62/101, 61.4%), followed by safety and harm (52/101, 51.5%).

In this section, we reflect on our findings through the lens of the 4 bioethical principles [117], while summarizing key results and highlighting research gaps. At the outset, we should clarify that we do not propose this framework as the sole or definitive approach and encourage further debate from diverse ethical perspectives. Rather, we use this familiar framework to indicate how bioethicists could think about the different misgivings we have articulated earlier. A further point to note is that we use the terms human supervision and human oversight interchangeably, referring collectively to the spectrum of involvement a human practitioner may have in overseeing CAI.

#### Nonmaleficence

Concerns related to nonmaleficence are about imposing harm, which mostly relate to the theme—safety and harm. Examples are concerns about the constant availability of CAI, which could potentially lead to overdependence and social isolation and about CAI making harmful and wrong suggestions. Human oversight may help mitigate the chances of such harm occurring. However, these risks are not exclusive to CAI. Humans can also cause harm, for example, due to time pressure or inappropriate interactions. This raises an important question: is harm caused by CAI somehow worse or more worrisome than harm caused by human practitioners?

#### Beneficence

The principle of beneficence requires that one ought to prevent harm, that one ought to remove evil or harm, and that one ought to do or promote good [117]. Building on the previous discussion, how might the use of CAI be viewed in terms of these duties of preventing or removing harm? One prominent concern is about crisis and suicidality management, for example, that CAI would respond inadequately to suicidality or other types of emergencies. The concern mentioned previously about CAI fostering social isolation could be interpreted as a failure to prevent the harm of social isolation. Meanwhile, some wonder whether CAI could play a helpful role in preventing harm, for instance, by being more approachable for some patients than traditional mental health crisis services.

Regarding the duty to promote good, a main expected benefit of CAI is that it could enhance the accessibility and availability of mental health support, potentially leading to better health outcomes. However, our review highlighted concerns that could undermine this potential, such as concerns about CAI’s effectiveness and its lack of empathy and humanness, which limit the extent to which it can promote good or *prevent* evil or harm. Failures in effectiveness are failures of beneficence since they are failures to promote patient health. We have identified three subthemes within this concern, worries about (1) the lack of clinical evidence; (2) CAI providers misrepresenting effectiveness, although, as we discuss in the subsequent sections, this misrepresentation can be understood as a failure to respect autonomy, and commercial CAI becoming more popular than effective CAI; and (3) worries about inherent effectiveness limitations, such as human-to-computer interactions not being translatable to human-to-human interactions. Many authors worried that because CAI’s simulated empathy differs from human empathy, this may affect engagement and therapeutic outcomes.

In response to these concerns, one countervailing consideration is that supervision may safeguard the effectiveness of care by offering the human side of care, such as genuine empathy, human therapeutic relationships and using transference and countertransference. In addition, the human professional could take adequate measures when the patient or others are in danger. Conversely, if supervision is not feasible and CAI use is therefore avoided, this could limit the potential to promote good, especially if CAI is shown to be effective in treating mental health issues.

#### Autonomy

Autonomy, one of the 4 principles of biomedical ethics, is the basis of concepts such as informed consent, truth-telling, and confidentiality [117,118]. While we classified autonomy as a separate theme, it spans several other themes such as explicability, transparency and trust, privacy and confidentiality, and concerns about the anthropomorphizing effects of CAI.

CAI’s algorithms are often considered opaque or a “black box.” This lack of transparency may hinder users’ understanding of CAI’s limitations and conceal potential hidden agendas of CAI companies. It may also hinder health care professionals, researchers, and regulators from independently verifying claims made by developers, including evaluating safety and security. In addition, it may also undermine patients’ informed choices and result in distrust in CAI and potentially in general mental health care as well. An open question remains: how much understanding of CAI’s mechanisms is necessary for patients to make informed choices and trust it?

Within the theme anthropomorphization and deception, misgivings arise about users anthropomorphizing CAI, despite their awareness of its nonhuman nature. Some authors worried that this can lead to deception, particularly if users are unaware of their tendency to anthropomorphize. Potential harms of this deception include user frustration, anxiety, violations of trust and autonomy, and ultimately reduced human interaction. Other authors have concerns about the potential erosion of shared decision-making if CAI bases recommendations on assumed, rather than actual, patient values, and concerns around coercion and manipulation because users are sometimes more compliant with CAI than humans.

Whether the anthropomorphizing features of CAI should be considered deceptive, manipulative, or coercive and therefore an obstacle to patient autonomy is something that needs further study. For example, should CAI truly be regarded as deceptive, manipulative, or coercive if patients know they are talking to CAI? Can CAI genuinely coerce given that it cannot straightforwardly carry out threats or coercive offers? Do these worries arise in a way that differs from similar concerns about deception, manipulation, or coercion when treatment involves human therapists?

We have also distinguished 3 subthemes regarding privacy and confidentiality, each of which are often justified by appealing to the principle of respect for autonomy [117]. The first is about how privacy is protected and regulated in current chatbots. Commercially accessible chatbots must adhere to different regulations than medical devices, which safeguard privacy and confidentiality differently. The second concerns the amount and types of data that CAI can collect and store. CAI differs from other eHealth interventions in the amount of data it collects, such as entire conversations, and the types of data it gathers (ie, when it uses smartphone sensors or use histories). These differences raise privacy concerns that are specific to CAI. The third subtheme compiles various potential harms related to privacy breaches—and thus brings the importance of preventing harm and the principles of beneficence and, potentially, nonmaleficence back to the fore. Such harms include emotional suffering and patients holding back information, thereby limiting the efficacy of treatment, and misuse of personal data when it gets into the hands of ill-intentioned persons or institutions.

#### Justice

Justice concerns in CAI primarily involve algorithmic bias, inequalities such as the digital divide, and epistemic injustice. CAI may, in certain ways, perpetuate or exacerbate inequalities. However, a main expected benefit of CAI is its accessibility and affordability, which may allow users without access to human professionals to receive some form of support, potentially reducing health inequalities. Even if CAI does not provide as much benefit as human therapists, it may still be better than no support at all. This ties into broader debates about the acceptability of care that falls short of the gold standard, a complex topic that warrants further exploration within the context of CAI.

#### Broader Topics of Concern

Some concerns about CAI extend beyond the 4 principles, most notably concerns about responsibility and accountability. Most authors argue for human responsibility over CAI’s decisions. However, the literature lacks consensus on which human actors should bear responsibility and whether these actors are willing and competent to assume it. In addition, apprehensions arise regarding the responsibilities of commercial CAI providers, who provide consumer-accessible CAI without human mental health care workers’ involvement. There are concerns about whether CAI overemphasizes patients’ own responsibility for mental well-being. Parker et al [119] have pointed out that while mental health apps’ tendency to promote individual responsibility may suit many consumers, it risks transforming it into a moral imperative. This may underemphasize or deny the social determinants of health. Supervised CAI use could address accountability by ensuring a human agent is responsible for outcomes, but this raises broader ethical questions about how responsibility for mental health should be divided between patients and health care professionals.

While this review focused on the ethical dimensions, questions about responsibility and accountability are connected to legal discussions. For instance, the responsibilities of clinicians versus software designers on the recommendation of CAI will differ between jurisdictions and individual circumstances. Further study into the legal implications of CAI use in mental health care is needed.

Other concerns that arguably extend beyond the 4 principles that warrant further exploration include the environmental impact of LLMs and concerns about the jobs of health care workers.

Finally, there are some additional research gaps, such as that our findings included relatively few empirical studies. Out of 101 included articles, only 9.9% (n=10) conducted empirical research. Especially, the perspectives and experiences of mental health patients are underexplored. Furthermore, we found that the lack of humanness is primarily mentioned in empirical studies among stakeholders and not discussed much in other publication types. Only one article addressed the theme of environmental impact—especially concerning climate change—of LLMs, despite media attention on its significance as a potential limitation [120].

### Suggestions for Future Research

On the basis of our review, identified research gaps and literature recommendations, the following avenues warrant exploration.

First, evaluations should be made on the risks and benefits of CAI in mental health care to determine whether its use is justifiable, even in principle. Research should clarify the roles of CAI and human practitioners and whether and how these two should be effectively integrated. Comparative analyses of CAI and human practitioners in supervised and unsupervised contexts are essential, including studies on the absence of human qualities in CAI and their influence on the therapeutic relationship and outcomes. Conversely, CAI could help study whether certain human therapist traits negatively affect treatment outcomes.

Focused analyses should address responsibility for CAI recommendations and the responsible use of training data. Regulations should define therapist responsibilities when patients use CAI outside of the consultation room. Understanding how various CAI uses, whether supervised or unsupervised, impact access to mental health care is essential, for ensuring justice and preventing inequalities. This includes whether CAI falls short of the gold standard of care, and if so, how this should affect its use. Also, examining the environmental impact of CAI, particularly LLMs, is crucial to balance their potential benefits with ecological harms.

Finally, empirical bioethics could enhance normative reflections on CAI use in mental health care [121]. This requires further empirical studies to explore stakeholder perspectives. For example, how do professionals perceive being held accountable for CAI’s output, and would they trust CAI without direct supervision? What do patients think of the simulated empathy of CAI, and do they feel deceived by anthropomorphic features? Answering these questions is essential for conducting normative analyses to inform the development of guidelines on the responsible use of CAI in mental health care.

### Strengths and Limitations

This scoping review is the first to specifically examine ethical issues in CAI for mental health care, making it timely and relevant amid rapid advancements in this field. Unlike narrative reviews, our study is distinguished by an extensive and interdisciplinary literature search. We conducted searches across multiple databases and disciplines following the recommendation for collaboration between biomedical experts and computer researchers in developing new AI applications for mental health care to avoid biases that arise due to the isolation of researchers within their respective disciplines [122]. Finally, this review provides a comprehensive overview of the quantity and types of ethical concerns, and its descriptive nature serves as a foundation for future research that addresses the practical and normative implications of these ethical considerations.

However, several limitations must be acknowledged. Methodologically, our focus on CAI in mental health care may have overlooked relevant ethical considerations in other AI health care applications. In addition, we concentrated on ethical dimensions, while legal aspects, particularly regarding accountability, are also important. Finally, the lack of consensus on terminology may have led us to overlook articles using alternative terms for CAI, although it remains uncertain whether this would have revealed additional ethical themes.

### Conclusions

This scoping review has investigated the ethical concerns raised in the literature about using CAI in mental health care. Ten main ethical themes were identified, with concerns about privacy and confidentially and safety and harm expressed most often. In addition, concerns specific to the use of conversational agents include the perceived lack of empathy and the worry of CAI replacing human-to-human contact and leading to social isolation. We found that a relatively small percentage of the articles (10/101, 9.9%) used empirical data collection methods and that the perspectives of certain stakeholders, especially patients with mental health disorders, are underrepresented.

We further observed issues needing more study, such as responsibility for CAI’s output, the potential limitations of CAI not being human and how these weigh against potential limitations of human therapists being human, how CAI use may impact inequalities in mental health care, and the environmental impact of AI. While the literature provides various potential solutions and recommendations to address some of the concerns, our review highlights the lack of empirical data and normative recommendations for using CAI in mental health care, signaling opportunities for future research. This review serves as a foundation for further normative analysis and the development of ethical guidelines on the responsible use of CAI in mental health care.

## Appendix

Multimedia Appendix 1 PRISMA-ScR (Preferred Reporting Items for Systematic Reviews and Meta-Analyses extension for Scoping Reviews) checklist.

Multimedia Appendix 2 Search strategy for the identification of articles.

Multimedia Appendix 3 Overview results of the identified articles.

## Abbreviations

AI: artificial intelligence
CAI: conversational artificial intelligence
LLM: large language model
PRISMA: Preferred Reporting Items for Systematic Reviews and Meta-Analyses
PRISMA-ScR: Preferred Reporting Items for Systematic Reviews and Meta-Analyses extension for Scoping Reviews

## Data Availability

The datasets other than Multimedia Appendices 1-3 that are generated during and analyzed during this study (ie, the Rayyan screening software files and the data charting raw spreadsheet) are available from the corresponding author on request.
