# Supplementary material for: Exploring the Ethical Challenges of Conversational AI in Mental Health Care: Scoping Review
Source: JMIR Ment Health. 2025 Feb 21;12:e60432. doi: 10.2196/60432 (PMC11890142; doi:10.2196/60432)
Supplement: Multimedia Appendix 2 [file mental_v12i1e60432_app2.docx]

| **Database searched** | **Platform** | **Years of coverage** | **Records** | **Records after duplicates removed** |
| --- | --- | --- | --- | --- |
| PubMed | PubMed | 1946 – Present | 431 | 431 |
| Embase | Embase.com | 1971 - Present | 460 | 175 |
| APA PsycInfo | EBSCO | 1968 - Present | 162 | 67 |
| Web of Science | Clarivate | 1975 - Present | 449 | 121 |
| Scopus | Elsevier | 1970 - Present | 983 | 441 |
| Philosophers Index | OVID | 1940 - Present | 39 | 21 |
| ACM digital library | ACM | 1908 - Present | 255 | 254 |
| **Total** | | | 2.779 | 1.510 |

|  | PubMed |  |
| --- | --- | --- |
| Search | Query | Results |
| #4 | Search: #1 AND #2 AND #3 Sort by: Most Recent | [431](https://pubmed.ncbi.nlm.nih.gov/?term=%2315+AND+%233+AND+%234&sort=date) |
| #3 | Search: "Psychotherapy"[MeSH Terms] OR "Therapeutic Alliance"[MeSH Terms] OR "Psychiatry"[MeSH Terms:noexp] OR "psychotherap*"[Title/Abstract] OR "therapist*"[Title/Abstract] OR "psychiatr*"[Title/Abstract] OR "Patient–physician relationship*"[tiab] OR "Patient–therapist relationship*"[tiab] OR "Patient–psychotherapist relationship*"[tiab] OR "transference"[tiab] Sort by: Most Recent | [573,868](https://pubmed.ncbi.nlm.nih.gov/?term=%22Psychotherapy%22%5BMeSH+Terms%5D+OR+%22Therapeutic+Alliance%22%5BMeSH+Terms%5D+OR+%22Psychiatry%22%5BMeSH+Terms%3Anoexp%5D+OR+%22psychotherap%2A%22%5BTitle%2FAbstract%5D+OR+%22therapist%2A%22%5BTitle%2FAbstract%5D+OR+%22psychiatr%2A%22%5BTitle%2FAbstract%5D+OR+%E2%80%9CPatient%E2%80%93physician+relationship%2A%E2%80%9D%5Btiab%5D+OR+%E2%80%9CPatient%E2%80%93therapist+relationship%2A%E2%80%9D%5Btiab%5D+OR+%E2%80%9CPatient%E2%80%93psychotherapist+relationship%2A%E2%80%9D%5Btiab%5D+OR+%E2%80%9Ctransference%E2%80%9D%5Btiab%5D&sort=relevance) |
| #2 | Search: "Ethics"[Mesh] OR "ethics"[Subheading] OR "Privacy"[Mesh:NoExp] OR "Safety"[Mesh:NoExp] OR "Confidentiality"[Mesh:NoExp] OR "Principle-Based Ethics"[Mesh] OR "Personal Autonomy"[Mesh] OR "Social Justice"[Mesh] OR "Human Rights"[Mesh] OR "ethic*"[tiab] OR "autonom*"[tiab] OR "social justice"[tiab] OR "privacy"[tiab] OR "human right*"[tiab] OR "confidential*"[tiab]OR "safety"[tiab] Sort by: Most Recent | [1,366,188](https://pubmed.ncbi.nlm.nih.gov/?term=%22Ethics%22%5BMesh%5D+OR+%22ethics%22%5BSubheading%5D+OR+%22Privacy%22%5BMesh%3ANoExp%5D+OR+%22Safety%22%5BMesh%3ANoExp%5D+OR+%22Confidentiality%22%5BMesh%3ANoExp%5D+OR+%22Principle-Based+Ethics%22%5BMesh%5D+OR+%22Personal+Autonomy%22%5BMesh%5D+OR+%22Social+Justice%22%5BMesh%5D+OR+%22Human+Rights%22%5BMesh%5D+OR+%22ethic%2A%22%5Btiab%5D+OR+%22autonom%2A%22%5Btiab%5D+OR+%E2%80%9Csocial+justice%E2%80%9D%5Btiab%5D+OR+%E2%80%9Cprivacy%E2%80%9D%5Btiab%5D+OR+%E2%80%9Chuman+right%2A%E2%80%9D%5Btiab%5D+OR+%E2%80%9Cconfidential%2A%E2%80%9D%5Btiab%5DOR+%E2%80%9Csafety%E2%80%9D%5Btiab%5D&sort=relevance) |
| #1 | Search: "Artificial Intelligence"[Mesh:NoExp] OR "Natural Language Processing"[Mesh] OR "Robotics"[Mesh] OR "Natural Language Process*"[tiab] OR "Robot*"[tiab] OR "conversational agent*"[tiab] OR "chatbot*"[tiab] OR "embodied robot*"[tiab] OR "embodied artificial intelligence*"[tiab] OR "Embodied AI"[tiab] OR "AI-Based App*"[tiab] OR "conversational artificial intelligence"[tiab] OR "conversational AI"[tiab] OR "digital assistan*"[tiab] OR "human-computer interaction*"[tiab] OR "computer-human interaction*"[tiab] OR "relational agent*"[tiab] OR "Embodied conversational agent*"[tiab] OR "Embodied Intelligent Application*"[tiab] OR "direct-to-consumer app*"[tiab] OR "social interface*"[tiab] OR "social robot*"[tiab] OR "telerobot*"[tiab] OR "Robot assist*"[tiab] OR "humanoid robot*"[tiab] OR "cyborg*"[tiab] OR "cybernetic*"[tiab] OR "human–machine interaction*"[tiab] OR "machine-human interaction*"[tiab] OR "chatGPT"[tiab] OR "chat-GPT"[tiab] OR "gpt-3.5"[tiab] OR "gpt-4"[tiab] OR "large language model*"[tiab] OR “artificial intelligence”[tiab] Sort by: Most Recent | [184,375](https://pubmed.ncbi.nlm.nih.gov/?sort=date&term=%22Artificial+Intelligence%22%5BMesh%3ANoExp%5D+OR+%22Natural+Language+Processing%22%5BMesh%5D+OR+%22Robotics%22%5BMesh%5D+OR+%E2%80%9CNatural+Language+Process%2A%22%5Btiab%5D+OR+%22Robot%2A%22%5Btiab%5D+OR+%22conversational+agent%2A%22%5Btiab%5D+OR+%22chatbot%2A%22%5Btiab%5D+OR+%22embodied+robot%2A%22%5Btiab%5D+OR+%22embodied+artificial+intelligence%2A%22%5Btiab%5D+OR+%22Embodied+AI%22%5Btiab%5D+OR+%22AI-Based+App%2A%22%5Btiab%5D+OR+%22conversational+artificial+intelligence%22%5Btiab%5D+OR+%22conversational+AI%22%5Btiab%5D+OR+%E2%80%9Cdigital+assistan%2A%22%5Btiab%5D+OR+%22human-computer+interaction%2A%22%5Btiab%5D+OR+%22computer-human+interaction%2A%22%5Btiab%5D+OR+%22relational+agent%2A%22%5Btiab%5D+OR+%22Embodied+conversational+agent%2A%22%5Btiab%5D+OR+%22Embodied+Intelligent+Application%2A%22%5Btiab%5D+OR+%22direct-to-consumer+app%2A%22%5Btiab%5D+OR+%E2%80%9Csocial+interface%2A%E2%80%9D%5Btiab%5D+OR+%E2%80%9Csocial+robot%2A%E2%80%9D%5Btiab%5D+OR+%E2%80%9Ctelerobot%2A%E2%80%9D%5Btiab%5D+OR+%E2%80%9CRobot+assist%2A%E2%80%9D%5Btiab%5D+OR+%E2%80%9Chumanoid+robot%2A%E2%80%9D%5Btiab%5D+OR+%E2%80%9Ccyborg%2A%E2%80%9D%5Btiab%5D+OR+%E2%80%9Ccybernetic%2A%E2%80%9D%5Btiab%5D+OR+%E2%80%9Chuman%E2%80%93machine+interaction%2A%E2%80%9D%5Btiab%5D+OR+%E2%80%9Cmachine-human+interaction%2A%E2%80%9D%5Btiab%5D+OR+%E2%80%9CchatGPT%E2%80%9D%5Btiab%5D+OR+%E2%80%9Cchat-GPT%E2%80%9D%5Btiab%5D+OR+%E2%80%9Cgpt-3.5%E2%80%9D%5Btiab%5D+OR+%E2%80%9Cgpt-4%E2%80%9D%5Btiab%5D+OR+%E2%80%9Clarge+language+model%2A%E2%80%9D%5Btiab%5D+OR+%E2%80%9Cartificial+intelligence%E2%80%9D%5Btiab%5D) |

Key: [Mesh]= medical subject heading, [tiab] = title, abstract, author supplied keywords.

|  | Embase (Embase.com) |  |
| --- | --- | --- |
| No. | Query | Results |
| **#5** | **#4** AND (**'article'**/it OR **'article in press'**/it OR **'editorial'**/it OR **'letter'**/it OR **'note'**/it OR **'review'**/it OR **'short survey'**/it) | **460** |
| **#4** | **#1** AND **#2** AND **#3** | **594** |
| **#3** | **'psychotherapy'**/exp OR **'therapeutic alliance'**/exp OR **'psychiatry'**/de OR **'psychotherap*'**:ab,ti,kw OR **'therapist*'**:ab,ti,kw OR **'psychiatr*'**:ab,ti,kw OR **'patient–physician relationship*'**:ab,ti,kw OR **'patient–therapist relationship*'**:ab,ti,kw OR **'patient–psychotherapist relationship*'**:ab,ti,kw OR **'transference'**:ab,ti,kw | **845,669** |
| **#2** | **'ethics'**/exp OR **'privacy'**/de OR **'safety'**/de OR **'confidentiality'**/exp OR **'personal autonomy'**/de OR **'social justice'**/exp OR **'human rights'**/exp OR **'ethic*'**:ab,ti,kw OR **'autonom*'**:ab,ti,kw OR **'social justice'**:ab,ti,kw OR **'privacy'**:ab,ti,kw OR **'human right*'**:ab,ti,kw OR **'confidential*'**:ab,ti,kw OR **'safety'**:ab,ti,kw | **2,091,458** |
| **#1** | **'artificial intelligence'**/de OR **'ambient intelligence'**/exp OR **'natural language processing'**/exp OR **'robotics'**/de OR **'natural language process*'**:ab,ti,kw OR **'robot*'**:ab,ti,kw OR **'conversational agent*'**:ab,ti,kw OR **'chatbot*'**:ab,ti,kw OR **'embodied robot*'**:ab,ti,kw OR **'embodied artificial intelligence*'**:ab,ti,kw OR **'embodied ai'**:ab,ti,kw OR **'ai-based app*'**:ab,ti,kw OR **'conversational artificial intelligence'**:ab,ti,kw OR **'conversational ai'**:ab,ti,kw OR **'digital assistan*'**:ab,ti,kw OR **'human-computer interaction*'**:ab,ti,kw OR **'computer-human interaction*'**:ab,ti,kw OR **'relational agent*'**:ab,ti,kw OR **'embodied conversational agent*'**:ab,ti,kw OR **'embodied intelligent application*'**:ab,ti,kw OR **'direct-to-consumer app*'**:ab,ti,kw OR **'social interface*'**:ab,ti,kw OR **'social robot*'**:ab,ti,kw OR **'telerobot*'**:ab,ti,kw OR **'robot assist*'**:ab,ti,kw OR **'humanoid robot*'**:ab,ti,kw OR **'cyborg*'**:ab,ti,kw OR **'cybernetic*'**:ab,ti,kw OR **'human–machine interaction*'**:ab,ti,kw OR **'machine-human interaction*'**:ab,ti,kw OR **‘chatGPT’**:ab,ti,kw OR **‘chat-GPT’**:ab,ti,kw OR **‘gpt-3.5’**:ab,ti,kw OR **‘gpt-4’**:ab,ti,kw OR **‘large language model*’**:ab,ti,kw OR **‘artificial intelligence’**:ab,ti,kw | **238,479** |

Key: Ab, ti, kw searches in abstract, title and author supplied keywords, /exp searches Emtree preferred indexing term, it=publication type

|  | **APA PsycInfo (EBSCO)** |  |  |
| --- | --- | --- | --- |
| **#** | **Query** | **Limiters/Expanders** | **Results** |
| S5 | S1 AND S2 AND S3 | Limiters – Academic journals | 162 |
| S4 | S1 AND S2 AND S3 | Search modes - Boolean/Phrase | 207 |
| S3 | DE "Psychotherapy" OR DE "Adlerian Psychotherapy" OR DE "Adolescent Psychotherapy" OR DE "Affirmative Therapy" OR DE "Analytical Psychotherapy" OR DE "Autogenic Training" OR DE "Brief Psychotherapy" OR DE "Brief Relational Therapy" OR DE "Child Psychotherapy" OR DE "Client Centered Therapy" OR DE "Conversion Therapy" OR DE "Couples Therapy" OR DE "Eclectic Psychotherapy" OR DE "Emotion Focused Therapy" OR DE "Existential Therapy" OR DE "Experiential Psychotherapy" OR DE "Expressive Psychotherapy" OR DE "Eye Movement Desensitization Therapy" OR DE "Feminist Therapy" OR DE "Geriatric Psychotherapy" OR DE "Gestalt Therapy" OR DE "Group Psychotherapy" OR DE "Guided Imagery" OR DE "Humanistic Psychotherapy" OR DE "Hypnotherapy" OR DE "Individual Psychotherapy" OR DE "Insight Therapy" OR DE "Integrative Psychotherapy" OR DE "Interpersonal Psychotherapy" OR DE "Logotherapy" OR DE "Narrative Therapy" OR DE "Network Therapy" OR DE "Persuasion Therapy" OR DE "Primal Therapy" OR DE "Psychoanalysis" OR DE "Psychodrama" OR DE "Psychodynamic Psychotherapy" OR DE "Psychotherapeutic Counseling" OR DE "Psychotherapeutic Techniques" OR DE "Rational Emotive Behavior Therapy" OR DE "Reality Therapy" OR DE "Relationship Therapy" OR DE "Solution Focused Therapy" OR DE "Strategic Therapy" OR DE "Supportive Psychotherapy" OR DE "Transactional Analysis" OR DE "Adolescent Psychotherapy" OR DE "Multisystemic Therapy" OR DE "Child Psychotherapy" OR DE "Play Therapy" OR DE "Gestalt Therapy" OR DE "Empty Chair Technique" OR DE "Group Psychotherapy" OR DE "Encounter Group Therapy" OR DE "Therapeutic Community" OR DE "Humanistic Psychotherapy" OR DE "Client Centered Therapy" OR DE "Hypnotherapy" OR DE "Age Regression (Hypnotic)" OR DE "Ericksonian Psychotherapy" OR DE "Posthypnotic Suggestions" OR DE "Integrative Psychotherapy" OR DE "Schema Therapy" OR DE "Psychoanalysis" OR DE "Adlerian Psychotherapy" OR DE "Brief Relational Therapy" OR DE "Dream Analysis" OR DE "Self-Analysis" OR DE "Psychotherapeutic Counseling" OR DE "Family Therapy" OR DE "Psychotherapeutic Techniques" OR DE "Active Listening" OR DE "Animal Assisted Therapy" OR DE "Autogenic Training" OR DE "Brief Relational Therapy" OR DE "Centering" OR DE "Cotherapy" OR DE "Dream Analysis" OR DE "Empty Chair Technique" OR DE "Ericksonian Psychotherapy" OR DE "Free Association" OR DE "Guided Imagery" OR DE "Life Review" OR DE "Mirroring" OR DE "Morita Therapy" OR DE "Motivational Interviewing" OR DE "Mutual Storytelling Technique" OR DE "Network Therapy" OR DE "Paradoxical Techniques" OR DE "Psychodrama" OR DE "Strategic Therapy" OR DE "Strategic Family Therapy" OR DE "Cognitive Therapy" OR DE "Online Therapy" OR DE "Therapeutic Alliance" OR DE "Psychotherapeutic Transference" OR DE "Psychiatry" OR TI("psychotherap*" OR "therapist*" OR "psychiatr*" OR “Patient–physician relationship*” OR “Patient–therapist relationship*” OR “Patient–psychotherapist relationship*” OR “transference”) OR AB("psychotherap*" OR "therapist*" OR "psychiatr*" OR “Patient–physician relationship*” OR “Patient–therapist relationship*” OR “Patient–psychotherapist relationship*” OR “transference”) OR KW("psychotherap*" OR "therapist*" OR "psychiatr*" OR “Patient–physician relationship*” OR “Patient–therapist relationship*” OR “Patient–psychotherapist relationship*” OR “transference”) | Search modes - Boolean/Phrase | 623,100 |
| S2 | DE "Consumer Ethics" OR DE "Ethics" OR DE "Secrecy" OR DE "Safety" OR DE "Social Justice" OR DE "Professional Ethics" OR DE "Privacy" OR DE "Privileged Communication" OR DE "Client Rights" OR DE "Human Rights" OR DE "Autonomy" OR TI("ethic*" OR "autonom*" OR “social justice” OR “privacy” OR “human right*” OR “confidential*” OR “safety”) OR AB("ethic*" OR "autonom*" OR “social justice” OR “privacy” OR “human right*” OR “confidential*” OR “safety”) OR KW("ethic*" OR "autonom*" OR “social justice” OR “privacy” OR “human right*” OR “confidential*” OR “safety”) | Search modes - Boolean/Phrase | 296,087 |
| S1 | DE "Natural Language Processing" OR DE "Human Robot Interaction" OR DE "Social Robotics" OR DE "Intelligent Agents" OR DE "Robotics" OR DE "Conversational Agents" OR DE "Artificial Intelligence" OR TI(“Natural Language Process*" OR "Robot*" OR "conversational agent*" OR "chatbot*" OR "embodied robot*" OR "embodied artificial intelligence*" OR "Embodied AI" OR "AI-Based App*" OR "conversational artificial intelligence" OR "conversational AI" OR “digital assistan*" OR "human-computer interaction*" OR "computer-human interaction*" OR "relational agent*" OR "Embodied conversational agent*" OR "Embodied Intelligent Application*" OR "direct-to-consumer app*" OR “social interface*” OR “social robot*” OR “telerobot*” OR “Robot assist*” OR “humanoid robot*” OR “cyborg*” OR “cybernetic*” OR “human–machine interaction*” OR “machine-human interaction*” OR “chatGPT” OR “chat-GPT” OR “gpt-3.5” OR “gpt-4” OR “large language model*” OR “artificial intelligence”) OR AB(“Natural Language Process*" OR "Robot*" OR "conversational agent*" OR "chatbot*" OR "embodied robot*" OR "embodied artificial intelligence*" OR "Embodied AI" OR "AI-Based App*" OR "conversational artificial intelligence" OR "conversational AI" OR “digital assistan*" OR "human-computer interaction*" OR "computer-human interaction*" OR "relational agent*" OR "Embodied conversational agent*" OR "Embodied Intelligent Application*" OR "direct-to-consumer app*" OR “social interface*” OR “social robot*” OR “telerobot*” OR “Robot assist*” OR “humanoid robot*” OR “cyborg*” OR “cybernetic*” OR “human–machine interaction*” OR “machine-human interaction*” OR “chatGPT” OR “chat-GPT” OR “gpt-3.5” OR “gpt-4” OR “large language model*” OR “artificial intelligence”) OR KW(“Natural Language Process*" OR "Robot*" OR "conversational agent*" OR "chatbot*" OR "embodied robot*" OR "embodied artificial intelligence*" OR "Embodied AI" OR "AI-Based App*" OR "conversational artificial intelligence" OR "conversational AI" OR “digital assistan*" OR "human-computer interaction*" OR "computer-human interaction*" OR "relational agent*" OR "Embodied conversational agent*" OR "Embodied Intelligent Application*" OR "direct-to-consumer app*" OR “social interface*” OR “social robot*” OR “telerobot*” OR “Robot assist*” OR “humanoid robot*” OR “cyborg*” OR “cybernetic*” OR “human–machine interaction*” OR “machine-human interaction*” OR “chatGPT” OR “chat-GPT” OR “gpt-3.5” OR “gpt-4” OR “large language model*” OR “artificial intelligence”) | Search modes - Boolean/Phrase | 39,397 |

Key: DE= Descriptors,TI= title, AB= abstract and KW= Searches for keywords in the uncontrolled content description of the document

|  | Web of Science (Core collection) - Clarivate |  |
| --- | --- | --- |
| Nr. | Query | Results |
| 4 | #1 AND #2 AND #3 | 449 |
| 3 | TS=("psychotherap*" OR "therapist*" OR "psychiatr*" OR “Patient–physician relationship*” OR “Patient–therapist relationship*” OR “Patient–psychotherapist relationship*” OR “transference”) | 516,803 |
| 2 | TS=("ethic*" OR "autonom*" OR “social justice” OR “privacy” OR “human right*” OR “confidential*” OR “safety”) | 2,126,818 |
| 1 | TS=(“Natural Language Process*" OR "Robot*" OR "conversational agent*" OR "chatbot*" OR "embodied robot*" OR "embodied artificial intelligence*" OR "Embodied AI" OR "AI-Based App*" OR "conversational artificial intelligence" OR "conversational AI" OR “digital assistan*" OR "human-computer interaction*" OR "computer-human interaction*" OR "relational agent*" OR "Embodied conversational agent*" OR "Embodied Intelligent Application*" OR "direct-to-consumer app*" OR “social interface*” OR “social robot*” OR “telerobot*” OR “Robot assist*” OR “humanoid robot*” OR “cyborg*” OR “cybernetic*” OR “human–machine interaction*” OR “machine-human interaction*” OR “chatGPT” OR “chat-GPT” OR “gpt-3.5” OR “gpt-4” OR “large language model*” OR “artificial intelligence”) | 441,844 |

Key: TS = topic, which includes title, abstract, author keywords and Web of Science Keywords Plus.

|  | Scopus (Elsevier) |  |
| --- | --- | --- |
| History Count | Search Terms | Results |
| 4 | #1 AND #2 AND #3 | 983 |
| 3 | TITLE-ABS-KEY ( "psychotherap*"  OR  "therapist*"  OR  "psychiatr*"  OR  "Patient--physician relationship*"  OR  "Patient--therapist relationship*"  OR  "Patient--psychotherapist relationship*"  OR  "transference" ) | 816,336 |
| 2 | TITLE-ABS-KEY ( "ethic*"  OR  "autonom*"  OR  "social justice"  OR  "privacy"  OR  "human right*"  OR  "confidential*"  OR  "safety" ) | 3,832,099 |
| 1 | TITLE-ABS-KEY(“Natural Language Process*" OR "Robot*" OR "conversational agent*" OR "chatbot*" OR "embodied robot*" OR "embodied artificial intelligence*" OR "Embodied AI" OR "AI-Based App*" OR "conversational artificial intelligence" OR "conversational AI" OR “digital assistan*" OR "human-computer interaction*" OR "computer-human interaction*" OR "relational agent*" OR "Embodied conversational agent*" OR "Embodied Intelligent Application*" OR "direct-to-consumer app*" OR “social interface*” OR “social robot*” OR “telerobot*” OR “Robot assist*” OR “humanoid robot*” OR “cyborg*” OR “cybernetic*” OR “human–machine interaction*” OR “machine-human interaction*” OR “chatGPT” OR “chat-GPT” OR “gpt-3.5” OR “gpt-4” OR “large language model*” OR “artificial intelligence”) | 1,563,074 |

Key: ABS, TITLE, AUTHKEY searches in abstract, title and author supplied keywords

|  | **Philosophers Index (OVID)** |  |
| --- | --- | --- |
| **#** | **Query** | **Results** |
| 1 | (Artificial Intelligence or Natural Language or Robotics or Robot or Machine Learning).sh. | 2.855 |
| 2 | (Embodied conversational agent* or Embodied Intelligent Application* or direct-to-consumer app* or social interface* or social robot* or telerobot* or Robot assist* or humanoid robot* or cyborg* or cybernetic* or human machine interaction* or machine-human interaction* or chatGPT or chat-GPT or gpt-3 or gpt-4 or large language model* or “artificial intelligence”)).mp.. [mp=abstract, title, heading word] | 4.056 |
| 3 | 1 or 2 | 4.694 |
| 4 | (Ethics or Privacy or Safety or Confidentiality or Autonomy or Social Justice or Human Rights).sh. | 125.6 |
| 5 | (ethic* or autonom* or social justice or privacy or human right* or confidential*OR safety).mp. [mp=abstract, title, heading word] | 158.041 |
| 6 | 4 or 5 | 158.232 |
| 7 | (Psychotherapy or Psychiatry).sh. | 2.974 |
| 8 | (psychotherap* or therapist* or psychiatr* or Patient physician relationship* or Patient therapist relationship* or Patient psychotherapist relationship* or transference or therapeutic alliance*).mp. [mp=abstract, title, heading word] | 4.857 |
| 9 | 7 or 8 | 4.857 |
| 10 | 3 and 6 and 9 | 39 |

Key: sh.= subject heading, mp.=abstract, title, heading word

|  | **ACM digital library (ACM)** |  |  |
| --- | --- | --- | --- |
| **#** | **Query** | **Filters** | **Results** |
| 1 | (“Natural Language Processing" OR Robot* OR "conversational agent" OR chatbot* OR "embodied artificial intelligence" OR "Embodied AI" OR "AI Based App" OR "conversational artificial intelligence" OR "conversational AI" OR “digital assistant" OR "human-computer interaction" OR "computer human interaction" OR "relational agent" OR "Embodied conversational agent" OR "Embodied Intelligent Application" OR "direct to consumer app" OR “social interface” OR “social robot” OR telerobot* OR “Robot assistant” OR “humanoid robot” OR cyborg* OR cybernetic* OR “human machine interaction” OR “machine human interaction” OR “chatGPT” OR “chat-GPT” OR “gpt 3.5” OR “gpt 4”OR “large language model” OR “artificial intelligence”) title AND (ethic* OR “personal autonomy” OR “social justice” OR privacy OR “human rights” OR confidential* OR safety) Anywhere AND (psychotherap* OR therapist* OR psychiatr* OR “Patient physician relationship” OR “Patient therapist relationship” OR “Patient psychotherapist relationship” OR transference OR “therapeutic alliance”) Anywhere | Journals | 255 |

Key: Title= title, Anywhere: searches in the all record content and in full-text
